# Fibrosis, the Bad Actor in Cardiorenal Syndromes: Mechanisms Involved

**DOI:** 10.3390/cells10071824

**Published:** 2021-07-19

**Authors:** Beatriz Delgado-Valero, Victoria Cachofeiro, Ernesto Martínez-Martínez

**Affiliations:** 1 Departamento de Fisiología, Facultad de Medicina, Instituto de Investigación Sanitaria Gregorio Marañón (IiSGM), Universidad Complutense de Madrid, 28040 Madrid, Spain; beadel02@ucm.es; 2 Ciber de Enfermedades Cardiovasculares (CIBERCV), Instituto de Salud Carlos III, 28029 Madrid, Spain

**Keywords:** cardiorenal syndrome, endoplasmic reticulum stress, fibrosis, heart failure, inflammation, kidney disease, oxidative stress

## Abstract

Cardiorenal syndrome is a term that defines the complex bidirectional nature of the interaction between cardiac and renal disease. It is well established that patients with kidney disease have higher incidence of cardiovascular comorbidities and that renal dysfunction is a significant threat to the prognosis of patients with cardiac disease. Fibrosis is a common characteristic of organ injury progression that has been proposed not only as a marker but also as an important driver of the pathophysiology of cardiorenal syndromes. Due to the relevance of fibrosis, its study might give insight into the mechanisms and targets that could potentially be modulated to prevent fibrosis development. The aim of this review was to summarize some of the pathophysiological pathways involved in the fibrotic damage seen in cardiorenal syndromes, such as inflammation, oxidative stress and endoplasmic reticulum stress, which are known to be triggers and mediators of fibrosis.

## 1. Introduction

The existence of a relationship between the heart and the kidney was first described in the XIX century by Robert Bright, who reported structural changes in the heart in patients with advanced kidney disease [1]. Since then, new discoveries have given insight into the interaction between heart and kidney diseases in terms of shared risk factors (such as hypertension, obesity, diabetes and atherosclerosis) and the pathophysiological pathways involved in each [2,3,4]. Clinically, the shared pathology of the heart and kidneys has a strong impact on the clinical outcome and is associated with increased morbidity and mortality rates [5,6].

The classic definition of cardiorenal syndrome (CRS) was proposed in 2010 by the Acute Dialysis Quality Initiative as a term that gathers the “disorders of the heart and kidneys whereby acute or chronic dysfunction in one organ may induce acute or chronic dysfunction of the other” [7]. In addition, within the term there is further classification into different subtypes according to the primary organ dysfunction and to whether it is an acute or chronic situation [7]. However, the appearance of risk factors that can affect both the heart and the kidney complicate the clinical picture, and with it the causal relationship of one to the other.

## 2. CRS Classification

### 2.1. CRS Type 1 or Acute Cardiorenal Syndrome

CRS type 1 (CRS-1) is characterized by the worsening of cardiac function leading to acute kidney injury (AKI) and/or dysfunction of both organs [7]. Around 25–30% of patients with acute decompensated heart failure (ADHF) present AKI, often after ischemic or non-ischemic heart disease [8,9,10]. These patients have higher morbi-mortality and lengthier hospitalization [7]. CRS-1 has a complex pathophysiology, with hemodynamic and non-hemodynamic alterations for which the treatments show no improvements [10,11], thus demonstrating the need to discover and understand the mechanisms involved.

Faced with a drop in blood pressure levels due to the development of heart failure (HF), the kidney responds to the decrease in cardiac output by retaining sodium and water. Nevertheless, it has been demonstrated that an elevation of the central venous pressure can result in impairment of renal function and congestion of the kidneys [10,12]. In this context, neurohormonal activation through the Renin–Angiotensin–Aldosterone System (RAAS) also has an important role, as it is both an initially compensatory mechanism for the decrease in volume consequence of the ventricular injury, and a long-term initiator of cardiovascular and renal dysfunction [13,14]. Other non-hemodynamic mechanisms, such as inflammation and oxidative stress, have been established as common pathways for cellular dysfunction in heart and kidney failure [9,10,11,15].

### 2.2. CRS Type 2 or Chronic Cardiorenal Syndrome

CRS type 2 is defined as chronic cardiac dysfunction that leads to progressive appearance of renal impairment that promotes the development of chronic kidney disease (CKD) [6,16,17]. CKD was defined in 2012 by Kidney Disease: Improving Global Outcomes (KDIGO) as an abnormality in kidney function or structure that is present for more than 3 months and has health implications. It is classified based on cause, a glomerular filtration rate (GFR) of <60 mL/min per 1.73 m^2^ and the degree of albuminuria [18]. A meta-analysis by Damman et al. showed that almost a third (32%) of the total of 1 million HF patients studied presented CKD, and 23% had worsening renal function [19], confirming that renal dysfunction is an important contributor to the comorbidities in HF.

The pathological process implicated in CKD secondary to HF is a consequence of the renal response to preserve the GFR. The combination of renal congestion, hypoperfusion and the increased right atrium pressure promotes renal dysfunction in HF patients [6,11]. It has been suggested that the correct diagnosis of this CRS should be based on HF aetiology, HF with preserved ejection fraction (HFpEF) or with reduced ejection fraction (HFrEF), and on biochemical parameters of renal dysfunction, such as creatinine levels [20]. However, as the interactions between the heart and kidney are bidirectional, is not always easy to assess the inciting event from the secondary damage, thus making it difficult to differentiate CRS type 2 patients from CRS type 4 ones [11,20].

### 2.3. CRS Type 3 or Acute Reno-Cardiac Syndrome

CRS type 3 occurs when there is an acute worsening of kidney function secondary to AKI, ischemia, or glomerulonephritis that leads to acute heart injury and/or dysfunction [6,7,11]. AKI may produce cardiac events as a consequence of the fluid overload, hyperkalaemia, or metabolic acidosis, but the exact cause of the damage is difficult to establish, as there are shared comorbidities and variability in the risk factors for AKI [6,11,21,22].

There are multiple definitions of AKI according to urine output and serum creatinine levels (SCr), all of which have limitations in their clinical application [21,23]. It is due to the differing definitions of AKI that make it difficult to identify this type of CRS. Despite the lacking criteria, the incidence of AKI is increasing in hospitalized patients, and is associated with an 86% increased risk of cardiovascular mortality and a 38% increased risk of major cardiovascular events [24].

### 2.4. CRS Type 4 or Chronic Reno-Cardiac Syndrome

CRS type 4 is characterized by cardiovascular damage in patients with CKD at any stage [7,11]. It is well established that renal dysfunction is an independent risk factor for cardiovascular disease, with the risk for myocardial infection and sudden death being higher in CKD patients [25,26]. Numerous studies have found there is an independent association between the severity of CKD, evaluated by the degree of decline in kidney function, and the subsequent cardiac events [5,27,28], which could suggest that CKD likely accelerates the risk and development of cardiovascular disease [7].

CKD has been demonstrated to be associated with inflammation and other cardiovascular factors, such as hypertension, activation of RAAS, or volume overload, that usually go in parallel with a decline in GFR [26,29]. Pressure and volume overload in CKD patients lead to left ventricular hypertrophy (LVH), which is a common feature that is accompanied by fibrosis and other histological changes. These structural changes consequently cause diastolic dysfunction and increased oxygen demand, which could also explain these patients’ predispositions to arrhythmias and sudden death [6,29,30].

### 2.5. CRS Type 5 or Secondary Cardiorenal Syndrome

CRS type 5 (CRS-5) represents simultaneous injury and/or dysfunction of the heart and kidneys as a result of a systemic condition, such as sepsis, drug toxicity, lupus, cirrhosis or amyloidosis [7,23,31]. Although many pathways have been proposed, it is challenging to identify the mechanisms that are involved in CRS-5 due to the multitude of contributing factors and the sequence of organ involvement [7,31].

CRS-5 has been divided into four stages according to severity: hyperacute (0–72 h after diagnosis), acute (3–7 days), subacute (7–30 days) and chronic (beyond 30 days) [6,23]. Usually, the existing studies of CRS-5 are those of hyperacute or acute stages, as these evaluate the effects of sepsis. Sepsis, defined as a life-threatening organ dysfunction caused by a deregulated host response to infection [32], is one of the most common causes of death among hospitalized patients [33], among whom the prevalence of CRS-5 is high [7,34].

In the early stages of sepsis, microcirculatory changes are developed despite normal systemic haemodynamics [35]. Those alterations, along with inflammation, are important in the cardiac and renal dysfunction given in this type of CRS [11]. For instance, the increase in pro-inflammatory cytokines during sepsis and the decrease in renal blood flow lead to tubular necrosis, reduction in GFR and severe kidney failure [6,23,26]. Sepsis is also related to autonomic nervous system dysfunction and RAAS activation [23,31]. This complex environment makes differentiating between the cardiorenal crosstalk effects and sepsis effects very difficult.

**Figure 1:**
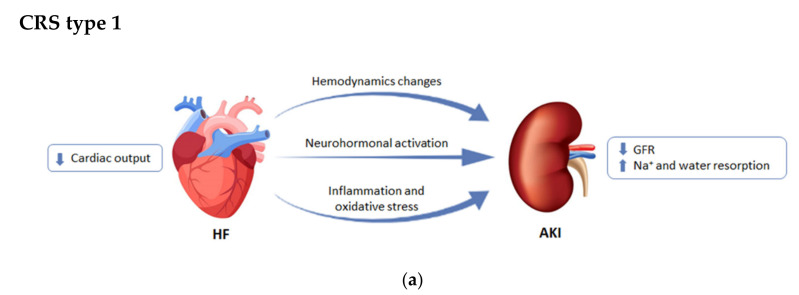

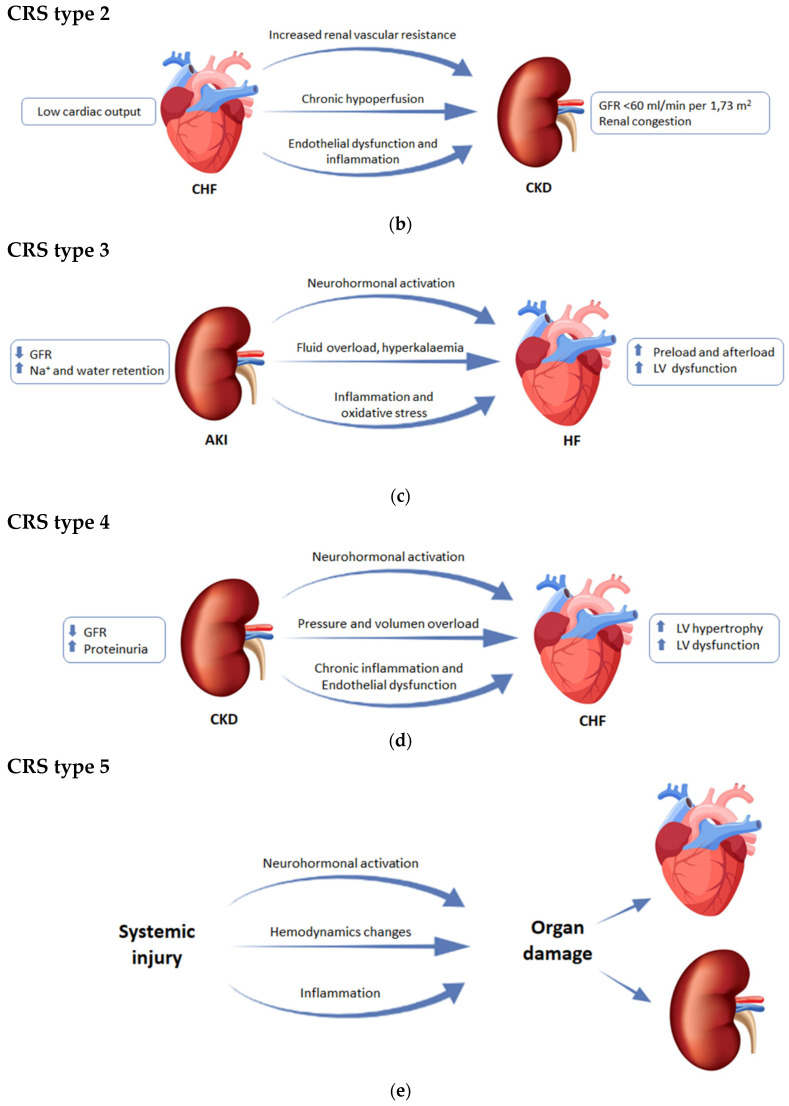
Differences among the subtypes of cardiorenal syndrome (CRS). (**a**) CRS type 1 or acute cardiorenal syndrome; (**b**) CRS type 2 or chronic cardiorenal syndrome; (**c**) CRS type 3 or acute reno-cardiac syndrome; (**d**) CRS type 4 or chronic reno-cardiac syndrome; (**e**) CRS type 5 or secondary cardiorenal syndrome. GFR: glomerular filtration rate; LV: left ventricular. Modified from [7].

The different CRSs are summarized in Figure 1.

## 3. Pathophysiology of CRS

Due to the essential role of both the heart and kidney in the maintenance of cardiovascular homeostasis, initial organ damage during a disease state, such as CRS, can induce structural remodelling and functional alterations in the other.

### 3.1. Cardiac Alterations Associated with CKD

As CKD is considered an important complication associated with higher cardiovascular risk and mortality. This increased risk is partially due to common risk factors such as hypertension, obesity or diabetes [36], but not entirely, as the association between CKD and cardiovascular mortality persists after risk factor adjustment [37,38]. Albuminuria- and creatinine-based estimated GFR (eGFR) are currently considered to be useful measurements for cardiovascular risk prediction, as they improve discrimination for cardiovascular mortality among CKD patients beyond traditional risk factors [38,39].

In patients with CKD there is high prevalence of structural and functional heart alterations from the early stages to end-stage renal disease (ESRD), which includes left ventricular (LV) remodelling, valvular sclerosis, reduction of the ejection fraction (EF) and diastolic dysfunction [40,41,42].

Echocardiographic studies have observed that LV remodelling is prevalent among patients with CKD and has been recognized as an important predictor of poor prognosis [43,44]. There are many factors that influence LV geometry in CKD patients. Pressure overload causes the thickening of the LV walls, which translates into concentric hypertrophy, whereas hypervolemia and anaemia contributes to the development of eccentric hypertrophy [45]. Two studies have reported the existence of associations between LV hypertrophy and renal dysfunction, characterized by low eGFR, which are independent of other risk factors, suggesting that impaired kidney function contributes to LV hypertrophy. In addition, they also describe that LV geometry tends to shift to concentric LV hypertrophy in advanced kidney dysfunction rather than eccentric hypertrophy [44,46]. A recent clinical study showed that the stages are associated with LV remodelling even in milder CKD, as 22% of 90 patients with stages 1 to 3 presented concentric hypertrophy, 19% eccentric hypertrophy and 20% concentric remodelling [47].

Most of the studies that have investigated the association between CKD and cardiac alterations have focused on the assessment of LV mass or hypertrophy, whilst fewer have explored LV function (neither systolic nor diastolic) [48]. In terms of LV systolic function, LVEF has been used in the majority of studies, although subclinical systolic dysfunction can happen in patients with CKD despite normal LVEF [49,50,51]. Diastolic dysfunction usually coexists with systolic dysfunction during LV remodelling and is common in CKD patients [44,52,53,54].

Numerous studies have assessed LV function in patients with CKD by trying to find an association between eGFR or albuminuria and systolic or diastolic function alterations. According to the literature, systolic dysfunction seems to be strongly correlated with albuminuria over low eGFR [55,56,57]. However, there is high variability. Similarly, there seems to be higher association of diastolic dysfunction with albuminuria than eGFR [56,57,58]. Therefore, some studies have found clear association between low eGFR, LV diastolic dysfunction and LVH [44,59].

Despite the advances made in cardiac damage, the increasing incidence and prevalence of HF makes it an important health problem. For that reason, various potential biomarkers that could contribute to diagnosis have been proposed. The gold standard in chronic HF diagnosis and prognosis is the natriuretic peptides, such as atrial natriuretic peptide (ANP) and brain natriuretic peptide (BNP), which are produced within the heart as a response to myocardial stretch as a consequence of volume or pressure overload [60,61]. HF guidelines currently recommends monitoring of BNP and its precursor, N-terminal-proBNP (NT-proBNP), for CHF progression evaluation. It must be acknowledged, however, that age, body mass, renal failure and pulmonary diseases influence its plasmatic concentrations [62]. Other molecules associated with myocyte necrosis or injury have been evaluated as HF biomarkers, such as cardiac troponins (cTn), which are regulatory proteins involved in contraction. The troponin complex is formed by cardiac troponin C (cTnC), I (cTnI) and T (cTnT), which dissociates after Ca^2+^ binds to cTnC. cTnI and cTnT are considered a reference marker of myocardial injury as its blood concentrations rise after myocyte damage [63,64].

### 3.2. Renal Alteration Associated to HF

As explained before, CRS-1 and CRS-2 are characterized by progressive kidney damage due to HF. Over 50% of HF patients have been reported to have renal insufficiency [19,65]. Indeed, even a modest reduction in renal function is associated with a higher mortality rate in cardiovascular disease patients [19,66]. The most currently used diagnostic measurements for renal damage are GFR, serum creatinine and urinary output.

The systolic blood pressure and effective arterial volume are reduced once HF develops, which translates into a decrease in renal blood flow as well as GFR [67]. In order to preserve adequate blood flow, the kidneys autoregulate through different mechanisms, including sympathetic nervous system (SNS) and RAAS activation, which would act as vasoconstrictors of the afferent and the efferent arteriole [13,68]. In the long term, this activation or the neurohormonal axis could result in podocyte injury [69,70], loss of mesangial integrity [71,72], tubular and glomerular damage [73,74,75] and kidney dysfunction [76], which are often associated with CKD and ESRD.

It is common to use the term kidney failure in a clinical setting to refer to a situation where there is a persistent decrease in eGFR in the short term [18]. Another important concept is worsening renal function, which is considered to appear in those patients in which the serum creatinine increases by 25% compared to the basal levels or the eGFR decreases by more than 20% in a period of around 25 weeks [77,78]. AKI is characterized by a rapid loss of kidney function that can happen in HF patients when diuresis decreases <0.5 mL/kg/h in 6–12 h or the basal serum creatinine levels increases ≥0.3 mg/dL in 48 h [77,78].

In addition to traditional markers of decreased glomerular filtration, such as creatinine and albuminuria [79,80], other markers, such as cystatin C [81,82] and blood urea nitrogen (BUN) [83,84], also have been proposed as possible biomarkers of tubular damage.

One of these is Neutrophil Gelatinase Associated Lipocalin (NGAL), a small glycoprotein expressed in renal and other cell types to which different functions have been attributed [85]. Its involvement in renal pathologies and its role as a biomarker comes from its rapid release in response to a tubular lesion and its presence in plasma, serum and urine, making it easy to quantify [85,86]. Another proposed molecule is kidney injury molecule-1 (KIM-1), a transmembrane glycoprotein expressed in low levels in healthy kidneys. Shortly after tubular damage, KIM-1 cleavage allows its secretion by the injured cells to the tubule lumen, resulting in detection in the urine, to where it is excreted [87]. Moreover, its role as a biomarker has proved to be associated with inflammation and fibrosis in the injured kidney, which would help monitor the degree of tubular damage [88,89,90]. Interleukin-18 (IL-18) is a proinflammatory cytokine that is expressed in activated macrophages, renal epithelial cells and others [91]. Urinary IL-18 is considered a marker of both short- and long-term injury in AKI, as it increases within 6 h of the insult or at least a day before serum creatinine increase [91,92].

### 3.3. Fibrosis

Another common structural alteration observed in both heart and kidney remodelling in CRS is fibrosis, which is also considered a key contributor to the progression of cardiac and renal failure [93,94,95]. Fibrosis is an important process that can be contemplated as aberrant wound healing as a consequence of the misbalance between extracellular matrix (ECM) production and degradation [96]. The fibrotic response to injury can be classified into reparative, when the scar is necessary to stabilize the tissue defect, or reactive, when the mechanical stress and the hormonal mediators facilitate the expansion of connective tissue in a remote non-injured zone, compromising the correct function of the organ [97]. The main fibrosis effectors are the fibroblasts and myofibroblasts, both of which are responsible for the synthesis and accumulation of interstitial ECM proteins. While fibroblasts are mesenchymal cells ubiquitous in tissues and organs, myofibroblasts are differentiated cells that are rarely found in non-pathological environments [98,99,100]. The fibrotic scar composition is similar amongst different tissues, predominantly formed by collagens type I and III, fibronectin, proteoglycans and laminin [101,102,103].

As a response to the damaged heart in cardiac ischemia, myocardial remodelling occurs through the secretion of ECM components by the myofibroblasts. Histopathologically speaking, there are three types of cardiac fibrosis: replacement fibrosis, interstitial fibrosis and perivascular fibrosis. Replacement fibrosis provides structural support, as it consists of the removal of necrotic tissue and generation of a fibrotic scar within the infarcted zone that compensates cardiomyocyte loss [104,105]. On the other hand, the widespread deposition of ECM proteins in the endo and perimysium of remote areas of the infarct is what is known as interstitial fibrosis [106]. The term perivascular fibrosis is used to describe the increase in connective tissue around the cardiac microvasculature [107], both of which are types of fibrotic lesions that could not be a consequence of cardiomyocyte death.

The remodelling that follows after MI happens in different phases that partially overlap: First, there is cell death and an inflammatory response (inflammatory phase); secondly, the resolution of inflammation and fibroblast proliferation (proliferative or reparative phase); and lastly, the scar formation and maturation (maturation or remodelling phase) [108]. During the proliferative phase, which usually coexists with the inflammatory and reparative phases, there is an increase in the number of fibroblasts, which will adopt the proliferatory, secretory and migratory myofibroblast phenotype [109]. Following the proliferative phase of cardiac repair, when the scar has been synthesized, there begins a long process known as maturation, in which an organized fibrotic state is formed due to ECM crosslinking [110] and scar reinforcement by other components of the ECM, such as decorin [111,112] and perlecan [113,114]. In addition, during the maturation phase, the activated fibroblasts go through apoptosis and senescence [115]. The presence of a mature fibrotic scar ultimately leads to an increased ventricular stiffness that compromises cardiac output [116,117]. In addition to the impaired cardiac contractility, fibrosis also interferes with the normal electrical signals within the heart, which predisposes to arrhythmias and fibrillation [118,119]. Overall, fibrosis has thus been proposed as a risk factor in HF as it predisposes to ventricular systolic and diastolic dysfunction [120,121,122], cardiomyocyte hypertrophy [122,123,124] and sudden cardiac death [125,126], thereby increasing mortality [127,128].

At the renal level, CKD is characterized by functional loss and deposition of connective tissue that ends up creating a common fibrotic phenotype independently of the initial damage. This happens since tubulointerstitial diseases lead to glomerular injury, and glomerular lesions produce tubulointerstitial damage. Fibrosis is a common manifestation of functional alterations that spreads in response to sustained inflammation and epithelial damage [129,130,131]. Among the events that induce fibrosis, both diabetes and hypertension are considered to be the leading causes of CKD [132,133], as they elevate the glomerular pressure that gradually leads to glomerular damage, endothelial dysfunction [134,135] and other structural changes, such as alterations of the glomerular basement membrane [136,137,138], decrease in podocyte number and mesangial distension [136,139,140]. As a result of such damage, the renal tissue would start a response that resembles wound healing in other tissues. The scar created in the early stage is potentially reversible but with the progression of the damage, the cross-linking of the ECM proteins makes it stiff and resistant to proteolysis [141].

During chronic injury to the kidney in CKD, the excessive accumulation of connective tissue and expansion of interstitial fibroblasts during the reparative stage of the fibrotic scar can happen in all compartments of the kidney, including the glomeruli, usually termed glomerulosclerosis, and the tubules, which is referred to as tubulointerstitial fibrosis [142,143,144]. Such deposition of the fibrotic matrix alters organ structure and function, which could further damage kidney function, as it impairs blood flow in this region of the parenchyma [96,145]. The fibrotic wound is not the only structural change involved since it is usually associated with tubular atrophy, tubular dilation and inflammatory cell infiltration [146,147,148]. Indeed, as the loss of renal cells and its replacement by ECM are common sequelae of renal damage, expansion of cortical fibrosis is considered one of the best histologic predictors of kidney dysfunction loss in CKD along with tubular atrophy (IFTA parameter) [148,149,150]. It is also one of the most common features assessed in biopsies in predicting a progression to ESRD [151,152].

Even though chronic damage to the kidney will naturally converge into histological and functional alterations that are common and lead to glomerulosclerosis and fibrosis, it is important to understand that the fibrotic progression is different depending on where it begins [153]. In glomerular damage, the progression starts with an injury within the Bowman’s Capsule that initially leads to glomerular hyperfiltration for a long period of time until it progresses to decrease the total GFR [154,155]. This reduction in the blood flow results in tubular hypoxia and epithelial cell death normally referred to as tubule atrophy [156,157]. In these circumstances, the inflammation initiated by the damaged tubular cells propitiates the formation of a fibrotic scar to fill the void created by epithelial cell death [158,159]. To form that scar, resident fibroblasts differentiate into the myofibroblast phenotype, which can synthesize different extracellular matrix proteins. Among the ECM components produced by myofibroblasts in order to form the fibrotic scar, the main ones in the kidney are collagen type I, III and IV, as well as fibronectin [160,161,162]. During tubule atrophy, the tubular basement membrane remains, thereby separating the cell death from the interstitium but disappears after the cell-free tubule collapses, at which point we could talk of complete loss of the nephron [163,164,165].

Epithelial damage is heterogeneous in tubular injury, which can be caused by many factors, such as hemodynamic, inflammatory, toxin-related or metabolic alterations. Some cells will instantly go through necrosis or apoptosis, whereas others will survive with different levels of injury, these being the ones that could proliferate and replace the lost cells of the tubular epithelium [166,167,168]. In the cases in which the tubules do not recover, inflammation signalling activates and with it the fibroblasts differentiate into myofibroblasts that will lead to tubulointerstitial fibrosis and tubular atrophy [169,170,171]. Tubulointerstitial fibrosis is the deposition of ECM proteins in the space between the tubular basement membrane and the peritubular capillaries [160], which impairs blood flow and induces ischemic injury in the nephrons of the fibrotic wound [148,172,173].

Inflammation and oxidative stress serve as the initial response to injury although its long-term progression could damage organ structure and function [174,175]. Inflammation is a common process in fibroproliferative diseases that leads to the release of pro-inflammatory mediators that have an important role in tissue damage and could either stimulate or inhibit fibrosis [176,177]. An appropriate level of cytokines and growth factors that mediates the cellular responses is key in normal wound healing. Among the many growth factors involved, transforming growth factor ß (TGF-ß) is considered to be a prototypic profibrotic cytokine that has a central role in organ fibrosis as it binds to its receptors causing the phosphorylation of SMADs, which modulate the expression of the target genes [100,178]. TGF-ß can also activate SMAD-independent pathways in what is called non-canonical signalling [179]. Among the many TGF-ß-mediated responses are cell proliferation and differentiation, ECM production and immune modulation [180,181,182]. Another important mediator is the connective tissue growth factor (CTGF), a downstream factor of TGF ß that has been reported in fibrosis in different organs such as the heart and kidney [93,183,184]. CTGF promotes the TGF-ß-induced excessive ECM production and fibroblast proliferation [185,186], and its expression appears to correlate with the degree of fibrosis [187].

As previously said, a dynamic balance between production and breakdown of ECM regulates the degree of fibrosis. The degradation of the ECM components is performed by the matrix metalloproteinases (MMPs), whose activity is controlled by the tissue inhibitors of MMPs (TIMPs) in order to maintain the homeostasis. MMPs can be classified according to substrate specificity into collagenases, such as MMP-1, MMP-8 and MMP-13 [188,189]; gelatinases, such as MMP-2 and MMP-9 [190,191]; membrane MMPs, such as MMP-14 [192]; and stromelysins, such as MMP-3, MMP-10 and MMP-11 [193]. Interestingly, MMPs can have both inhibitory and stimulatory effects on fibrosis as some of them promote it [194]. For example, the most frequently studied MMPs in HF and kidney damage are MMP-2 and MMP-9, out of which MMP-9 is believed to have a profibrotic effect [195,196,197] whereas MMP-2 has antifibrotic effects [198,199].

In recent years, it has been proved that different metabolic alterations stimulate structural and/or functional alterations, such as fibrosis development. Changes in metabolic regulation, such as that occurring in a situation such as lipotoxicity, defined as the accumulation of lipids in non-adipose tissues, is known to promote the development of fibrosis. This fibrosis is due to an upregulation in ECM protein synthesis, promoted by fibroblasts [200,201]. In this sense, we have observed in a recent study that MI is associated with cardiac lipotoxicity in rats, independently of the presence of obesity. This lipotoxicity was accompanied by alterations in the mitochondrial lipid profile and associated with myocardial fibrosis, suggesting that MI promotes an increase in lipid accumulation in the heart through mechanisms that are currently unknown. Similarly, we observed at the renal level the direct profibrotic role of palmitic acid at renal fibroblasts, as it induced an increase in ECM synthesis mediated by activation of ER stress, suggesting its importance in lipotoxicity-induced fibrosis [202]. These observations are in agreement with another study in which the authors proved that accumulation of lipid droplets accelerates tubulointerstitial fibrosis development in an animal model of kidney disease [200].

## 4. Mechanisms Involved in Fibrosis Progression

As a wide variety of diseases converge in fibrosis understanding, the pathogenesis involved is important in order to determine potential therapeutic targets. Despite the efforts to acquire insight into the process, the mechanisms involved are not fully established, and the current therapies are either ineffective or only slightly successful [203,204]. The current clinical strategies for CRS are guided towards the treatment of the general processes, such as diuretics, to treat volume overload, or angiotensin converting enzyme (ACE) inhibitors, Angiotensin II receptor blockers, mineralocorticoid receptor antagonist or β-adrenergic blockers to inhibit RAAS activation [17,205]. Due to the complex pathophysiology of CRS, new therapeutic approaches centred in fibrosis have been proposed. For instance, in a recent study, it has been proved that cardiac shock wave therapy significantly reduces cardiac fibrosis in a rat model of MI through the activation of the PI3K/Akt signalling pathway [206]. Despite this, these new experimental approaches are still required in order to have a comprehensive understanding of the pathophysiological mechanisms underlying fibrosis.

### 4.1. Inflammation

Inflammation can be defined as a defensive immune response that is triggered by damage to a tissue. The acute inflammatory response can be initiated as a consequence of an infection in which the pattern recognition receptors in the innate immune cells interact with the pathogen-associated molecular patterns (PAMPs), or due to the damage-associated molecular patterns (DAMPs) that are released during physical injury [207]. An acute inflammatory response is characterized by vasodilation, vascular leak and leukocyte emigration and, shortly after its induction, secretion of cytokines and chemokines will happen in order to recruit the immune cells to the damaged or infected region. Among the cells recruited, neutrophils are the first to migrate as a means to engulf the pathogens and secrete pro-inflammatory mediators and vasoactive substances [208,209].

In a normal inflammatory response, the activity is temporally restricted, as it resolves once the threat has been dealt with. However, the presence of a prolonged low-grade activity leads to chronic inflammation, which is characterized by the activation of different immune components that lead to major alterations in tissues, increasing the risk of diseases [210]. The clinical consequences of chronic inflammation include type 2 diabetes [211,212], hypertension [213], cardiovascular disease [214,215], chronic kidney disease [216]) and metabolic syndrome [217] among others.

Since both CHF and CKD are associated with a chronic inflammation response, characterized by an increase in the circulating inflammatory mediators, this process has become of interest in the understanding of CRS. A persistent inflammatory trigger is needed in order to activate the wound-healing process. However, if not eliminated quickly, the inflammatory cells could increase the response, leading to the abnormal wound healing and scarring characteristic of fibrosis. Within the wound-healing mechanism that is activated after injury, the first response is coagulation, in which activated platelets release platelet-derived growth factor (PDGF), acting as a chemoattractant for inflammatory cells, and transforming growth factor ß1 (TGF-ß1), which is one of the main drivers of fibrosis as it stimulates ECM synthesis by the fibroblasts of the tissue that was damaged [218,219,220].

Inflammation is known to have an important role in the development and progression of chronic diseases. For example, CKD progression into ESRD is characterized by chronic inflammation in the renal parenchyma, concluding in ECM deposition and loss of renal function [221,222,223]. Independent of the original cause, experimental models and human biopsies have shown that during renal inflammation, cells such as neutrophils and macrophages infiltrate both the glomeruli and tubulointerstitial space in order to remove the cell and matrix components that were damaged during the insult [223,224,225]. In general, M1 macrophages generate the initial response in the diseased organ by generation of pro-inflammatory cytokines, such as tumour necrosis factor α (TNFα) and interleukin-1 (IL-1), whereas M2 macrophages propitiate tissue repair by secretion of immunosuppressive cytokines during the repair phase [223,226]. It is that transition from the M1 to M2 phenotype that promotes fibrosis, as the production of cytokines, chemokines and growth factors alter the ECM balance between production and degradation [214,227,228].

Cytokines are cell-derived polypeptides that mediate the inflammatory response and can have positive or negative effects. It is well known that not all cytokines are involved at all stages of inflammation, but some of them do mediate both acute and chronic responses. This is the case of TNF-α, IL-1 (α and β) and IL-6 [229], which are some of the most studied ones and have been suggested to have an important role in inflammatory modulation during CRS due to its extremely potent proinflammatory effects [94,230,231,232].

It is well established that RAAS activation and the sympathetic nervous system (SNS) promotes the inflammatory response both in the heart and kidneys [233]. Angiotensin II (Ang II), one of the main effectors of RAAS activation, induces endothelial dysfunction, upregulation of adhesion molecules and fibrosis [234,235,236]. These Ang II effects are accompanied by recruitment of infiltrating cells and an increase in proinflammatory cytokines via the angiotensin type 1 (AT1) receptor in cardiorenal disease [230,237]. It has been proved that Ang II produces the accumulation of macrophage in the kidney [238,239], and it was shown in a murine unilateral ureteral obstruction (UUO) model that the macrophages’ AT1 receptor activation impedes polarization towards the M1 phenotype and limits the damage and fibrosis [240]. This shows that an increase in M1 macrophage differentiation makes organs more susceptible to damage whereas the M2 phenotype decreases injury [223,241,242]. Nevertheless, neurohormonal activation is not the only proposed source of inflammation in CRS. Both animal and human studies have shown that congestion may lead to endothelial activation and peripheral release of proinflammatory mediators, as venous congestion itself causes an inflammatory response activation in cells [233,243,244].

Inflammation leads to functional and structural damage in the cardiorenal axis, as the different cytokines, especially TNF-α, which plays a central role in organ dysfunction, are involved in inflammation, cell proliferation [245] and apoptosis [246]. During inflammation, TNF-α has been described to be involved in vasodilation, inflammatory cell adhesion, coagulation and reactive oxygen species (ROS) production, among others [247].

Numerous cytokines have been studied due to their profibrotic or antifibrotic effects [248]. Th2-derived cytokines, such as IL-4, IL-5, IL-6, IL-13 and IL-21, are important in the regulation of organ fibrosis [249,250], out of which the most studied one is IL-13, an interleukin whose profibrotic effect can be enhanced by IL-5 and IL-21, and which can increase its production and its receptor expression [251,252,253]. IL-21 can also promote tissue fibrosis through the induction of differentiation into Th17 cells [254,255], which produce a well-known profibrotic interleukin, IL-17, and which is involved in the development of fibrosis in various organs [256,257,258], although a recent study has suggested IL-17 plays an antifibrotic role in tubulointerstitial fibrosis [259]. On the other hand, Th1 cytokines, such as IL-7 [250,260], IL-10 [261,262], IL-12 [263,264] and IL-22 [265,266], along with IFN-γ [267,268], have been shown to have a suppressive effect on fibrosis. For instance, the inflammatory response in IL-10 KO mice resulted in scar formation rather than wound repair, suggesting IL-10 has an important antifibrotic role [269,270].

Chronic, unresolved inflammation damages renal structure and function, thereby leading to CKD, a state characterized by progressive renal fibrosis. In previous studies, it was reported that circulating levels of fibrinogen, TNF-α and a decrease in serum albumin were associated with loss of kidney function, linking the progression of CKD to the inflammatory response [221,271]. Systemic inflammation and function decline can alter the structure of the kidney, creating an environment in which epithelial damage increases and the factors released by infiltrating macrophages lead to fibrotic expansion [272,273]. Indeed, macrophage depletion has proved to reduce renal fibrosis in an animal model of myocardial infarction [274]. In renal fibrosis, the first process involved is the injury itself, followed by the unresolved inflammation. In the tubulointerstitium, pro-inflammatory cytokines, such as IL-6, TNF-α and IL-1β, promote further inflammatory cell infiltration, propitiating activation of profibrotic cells to differentiate into myofibroblasts and local secretion of fibrotic mediators [275,276,277]. This situation will lead to overproduction and deposition of ECM proteins, disruption of tissue integrity and progressive decline in function. Finally, glomerulosclerosis and tubular atrophy will happen in the latest stages [278,279].

In cardiac injury, as what happens in renal damage, the necrotic cell death within the heart activates tissue cells that will synthetize proinflammatory cytokines to recruit inflammatory cells. In the first phase, the macrophages and neutrophils act to remove the debris and release growth factors and cytokines that propitiate formation of connective tissue. Afterwards, fibroblast activation and cell proliferation will happen in the maturation phase to repair the myocardium by fibrotic wound formation [280,281]. After the phagocytic clearance of the apoptotic cells, macrophages will polarize towards the “reparative” M2 phenotype, releasing anti-inflammatory and profibrotic cytokines such as IL-10 and TGFβ, while proinflammatory cytokines, such as IL-1β or TNF-α, decrease in order to stimulate cardiac fibroblast activation to collagen-secreting myofibroblast [281,282,283]. Having said that, chronic inflammation entails a change in the inflammatory behaviour towards persistent and exacerbated fibrinogenesis, which is a structural feature in chronic injuries. It is due to that characteristic chronic inflammation for which TNF-α has been proposed as an independent predictor of cardiac and non-cardiac mortality in CHF patients [284]. Nonetheless, there is no consensus on the role of cytokines and chemokines, as some studies suggest its aggravating injury effects and others show that they endanger cardioprotective responses. For example, TNF-α ablation has proved to reduce the infarct size in mice with I/R injury [285], but in other studies TNF receptor deficiency increased the ischemic injury during I/R [286].

### 4.2. Oxidative Stress

Oxidative stress is a general concept that describes the imbalance between the production of ROS and the antioxidant defences. ROS includes both free radicals, which are species with an unpaired electron, such as superoxide anion (O_2_^•−^) and hydroxyl radical (∙OH), or non-free radical oxygenated molecules, such as hydrogen peroxide (H_2_O_2_) [287,288]. Other reactive species derived from nitrogen or sulphur do exist, but they are less abundant [289,290].

Even in basal conditions, aerobic metabolism involves ROS production, thus making O_2_^•−^ and H_2_O_2_ physiological intracellular metabolites. In low quantities, ROS act as signalling molecules involved in different pathways, such as cell proliferation, apoptosis and gene expression [291,292]. However, the fact that an important increase in oxidants could target almost all substrates implies the impairment and alteration of all biomolecules, resulting in cell damage and death [293,294]. ROS can damage proteins [295] and nucleic acids [296,297], but among all the molecules to undergo oxidation, polyunsaturated fatty acids are the most susceptible, leading to an increase in the markers of lipid peroxidation, such as malondialdehyde or 4-hydroxynonenal [298,299,300].

The endogenous sources of prooxidant species include organelles where there is high oxygen use, such as the mitochondria, peroxisomes, due to the fatty acid β-oxidation [301,302], and the endoplasmic reticulum (ER) [303], although the mitochondria seem to be the major source of ROS production, as around 95% of the breathed oxygen is reduced in the mitochondrial electron chain. Specifically, there are two major sites in the electron transport chain, the NADH dehydrogenase (complex I) and the ubiquinone cytochrome c reductase (complex III), which transfer electrons to coenzyme Q or ubiquinone, creating reduced forms that will ultimately transfer electrons to the molecular oxygen, generating superoxide radicals [304,305]. Through the action of mitochondrial superoxide dismutase (SOD), the superoxide anion is converted to hydrogen peroxide, which can be detoxified by the catalase and glutathione peroxidase [305,306].

In the outer mitochondrial membrane, the monoamine oxidases are another source of ROS that is not related to respiration [307,308]. In this case, the bivalent reduction of oxygen produces H_2_O_2_. In order to regulate the levels of ROS, the sources colocalize with the antioxidant response, among which there are enzymes, such as superoxide dismutases, catalase and glutathione peroxidase, as well as non-enzymatic antioxidants, such as vitamin A, bilirubin or reduced coenzyme Q [288,309,310].

Both inflammation and oxidative stress are related to chronic diseases, such as diabetes, hypertension, cardiovascular diseases or CKD [175,311,312,313]. It is known that under chronic damage the inflammatory and hypoxic environment propitiates fibrosis by fibroblast activation and proliferation into myofibroblasts. In this circumstance, ROS formation also occurs, and is considered to have an important role in both inflammation and organ fibrosis [314,315,316]. The bidirectional link between ROS and TGF-β1 is well established, as ROS production and enhanced ROS formation leads to higher activation and expression of TGF-β1 [317,318,319]. One of the possible explanations for this link resides in the action of an important ROS source, such as the different NADPH oxidases (NOX). In normal conditions, the NOX-derived ROS act as modulators of cell growth, proliferation, differentiation and apoptosis, but once it is uncontrolled, oxidative stress damages the DNA, proteins and lipids, inducing organ damage and fibrosis [320,321,322]. Multiple studies have shown the effectiveness of NOX-1 and NOX-4 inhibition in inflammation and fibrosis amelioration in liver and kidney injury [323,324,325], while different studies in the heart have shown that both NOX-2 and NOX-4 mediate the oxidative stress and cardiac injury following I/R [320,326,327]. Indeed, NOX-4 is considered a well-recognized mediator of the transition from fibroblast to myofibroblast, and its inhibition in in vitro studies with renal cells proved to prevent ROS production and myofibroblast differentiation, which would translate into a decrease in fibrosis during damage [328,329].

Multiple factors seem to participate in order to produce the characteristic multiorgan dysfunction of CRS, among which the increase in proinflammatory cytokines, the dysregulation of apoptosis and the increase in oxidative stress have been proposed as key elements of this complex pathophysiology [232,330,331]. Different animal models have shown that an increase in oxidative stress plays a pivotal role in cardiac and renal damage, independently of the CRS type depicted, through activation of the inflammatory response [15,331,332,333]. This can also be seen in patients with CRS, who presented an increase in ROS and RNS, which was accompanied by higher inflammatory cytokines, such as IL-6 [15].

### 4.3. Endoplasmic Reticulum Stress

The ER is an essential organelle for calcium homeostasis, lipid biosynthesis and protein synthesis and post-translational modifications. To ensure correct protein folding, the ER lumen balance between unfolded and misfolded proteins, and the capability to handle it, must be maintained. Such homeostasis could be altered by both physiological and pathological entities, such as inflammatory cytokines, protein demand or mutant protein expression, which translates into what is called ER stress [334,335].

In response to ER stress, the unfolded protein response (UPR) is initiated by at least one of three different pathways: the ER transmembrane proteins Activating Transcription Factor 6 (ATF6), Inositol-Requiring 1 (IRE1) or PKR-like ER kinase (PERK). In unstressed conditions, the chaperone Immunoglobin Binding Protein (BiP) binds to the luminal domain of ATF6, IRE1 and PERK, keeping them inactive [334,336]. In ER stress conditions, BiP dissociates from the three regulators, activating UPR [337]. Although initially UPR is considered a beneficial adaptive response, if it fails to restore homeostasis, then the UPR pathways guide the damaged cells to apoptosis and the consequent tissue injury [338,339].

Different pathologies, such as diabetes mellitus [340], obesity [341,342], cardiovascular disease [343,344] and CKD [345,346], have been associated with ER stress. In CRS, the activation of ER stress in the heart and kidney could be induced by different factors, such as hemodynamic changes, hormones from the RAAS, inflammation or oxidative stress [346,347,348]. These pathophysiological mediators could directly induce ER stress in the myocardium or renal parenchyma, resulting in apoptotic cell death due to prolonged UPR activation [349,350,351] and the consequent fibrotic wound formation, all of which would eventually lead to structural and functional changes [348,352,353,354]. Our group has recently evaluated the effect of myocardial infarction (MI) at renal level in rats. At 4 weeks post-MI, animals presented renal alterations characterized by tubulointerstitial fibrosis, oxidative stress and upregulation of inflammatory cytokines, such as IL-6 and TNF-α. All these alterations were accompanied by ER stress activation, which correlates with the renal fibrosis, suggesting ER stress relevance in the structural renal damage in CRS type 1 [202].

As ER stress inhibition has proved to ameliorate the fibrotic progression, it has been suggested that its blockade could be a new therapeutic approach for fibrosis [355,356,357]. One of the possible ways in which ER stress could lead to fibrosis is through fibroblast differentiation and collagen formation by TGF-β upregulation, as PERK and IRE1 activation have been seen to increase TGF-β expression [358,359,360]. ER stress activation of fibroblasts during injury at the wounded site triggers their differentiation into myoblasts, so as to restore the area by ECM protein synthesis and secretion [361,362]. Different in vitro studies have described ER-mediated differentiation into different cell types, such as renal tubular cells [363], cardiac cells [364], adipocytes [365,366], plasma cells [367,368] and others [369,370,371]. Additionally, our group’s in vitro studies in kidney fibroblasts, stimulated with the well-known profibrotic factor Ang II in presence of the pharmacological inhibitor of ER stress, 4-phenylbutiric acid (4-PBA), proved to be effective in preventing the increase in collagen I, inflammatory markers and superoxide anion production. All of this suggests the important role of ER stress in fibrosis, inflammation and oxidative stress in renal damage [202].

**Figure 2:**
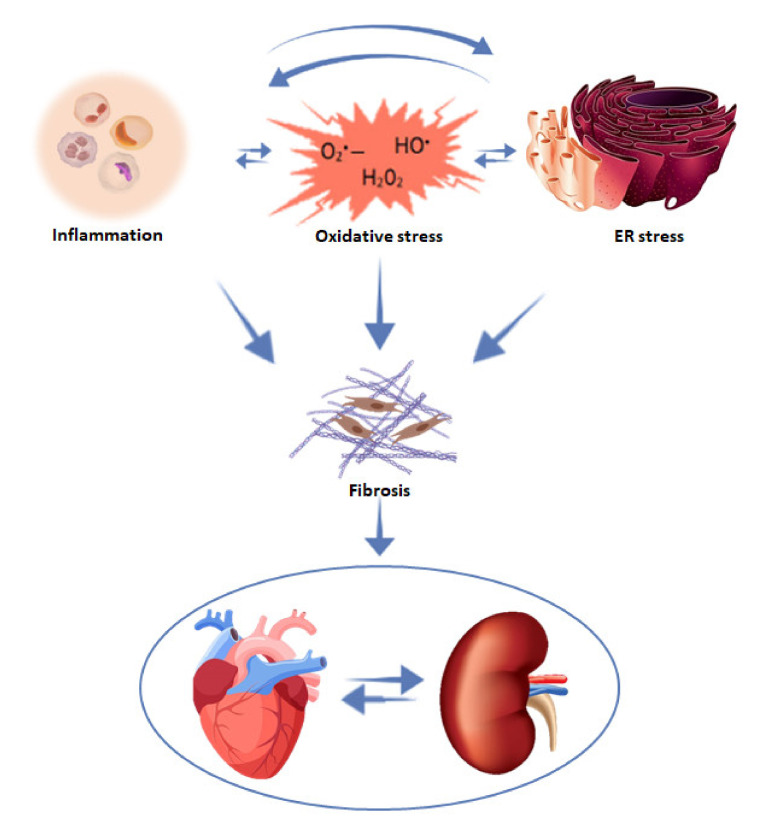
Mechanisms involved in the progression of cardiac and renal fibrosis in CRS.

The explained mechanisms involvement in CRS is depicted in Figure 2.

## 5. Conclusions

Due to the pathogenesis of cardiorenal syndromes, numerous efforts have given insight into the different pathways and mediators involved. This review has summarized evidence that the development of a fibrotic wound has proved to play a central role in both cardiac and renal damage progression, in which inflammation, oxidative stress and ER stress could be relevant players. This makes it crucial to understand the pathogenic basis of fibrosis in order to determine therapeutic targets.
